# Eternal nodules to fix the nitrogen issue: Promotion of soybean nodule senescence by a NAC/CYP module

**DOI:** 10.1093/plcell/koad154

**Published:** 2023-05-26

**Authors:** Nicolas M Doll

**Affiliations:** Assistant Features Editor, The Plant Cell, American Society of Plant Biologists, USA; Department of Plant Biotechnology and Bioinformatics, Ghent University, Ghent 9052, Belgium; VIB Center of Plant Systems Biology, Ghent 9052, Belgium

The nitrogen issue has 2 faces in today's agriculture: although nitrogen availability in soil is a serious limitation to crop production, the extensive use of chemical fertilizers for soil enrichment is harmful to the environment and humans. In this regard, legumes have the big advantage of fixing atmospheric nitrogen, allowing soil enrichment without the negative effects of chemical fertilizers. Thus, legumes could play a pivotal role in making the agriculture of tomorrow sustainable (Stagnari et al 2017).

Nitrogen fixation in legumes takes place in specialized root nodules and involves a symbiotic interaction with rhizobia bacteria from the soil. Inside the nodule, rhizobia differentiate into specialized bacteroids, which produce the nitrogenase enzyme that reduces atmospheric nitrogen to ammonia. As nitrogenase activity is inhibited by dioxygen, the host plant produces leghemoglobin that binds O_2_ and prevents its diffusion into the nodule. In return for the nitrogen fixed, the host plant provides photosynthates to the bacteroids. Nodules eventually senesce, allowing the plant to allocate resources to support other developmental processes. In senescent nodules, leghemoglobin levels and nitrogenase activity decrease and therefore progressively lose their nitrogen fixation potential (Puppo et al 2005). Consequently, delaying nodule senescence is a potential target for enhancing nitrogen fixation by legumes in the field.

In this issue, **Haixiang Yu and colleagues** (Yu et al. 2023) show that control of nodule senescence by members of the NAC transcription factors family impacts nitrogen fixation in soybean. Firstly, they identified 24 NACs upregulated during nodule senescence based on RNA-seq analysis. They selected 10 of them that are representative of all NAC subfamilies and overexpressed them in soybean roots. Interestingly, both *GmNAC039* and *GmNAC018* overexpression resulted in plants with yellowish leaves, indicative of nitrogen deficiency. These lines had significantly smaller root nodules. In addition to yielding fewer bacteroids, the overexpression lines had decreased expression of leghemoglobin genes and reduced nitrogenase activity; the nodules had degenerating nuclei with fragmented DNA, indicating early senescence. Conversely, senescent nodules of a *gmnac039 gmnac018* double knock-out mutant had higher nitrogenase activity and increased expression of leghemoglobin genes compared to the wild type, suggestive of delayed nodule senescence. To assess if *GmNAC039* and *GmNAC018* trigger nodule senescence by repressing or activating target genes, the authors fused either the VP16 or the SRDX domain to GmNAC039, thus turning this transcription factor into a constitutive transcriptional activator or repressor, respectively. They found that *GmNAC039-VP16* overexpression mimics *GmNAC039* overexpression, while GmNAC039-SRDX overexpression mimics *gmnac039 gmnac018* double mutant, indicating that GmNAC039 acts as a transcriptional activator of genes promoting nodule senescence (see Fig.).

**Figure.**
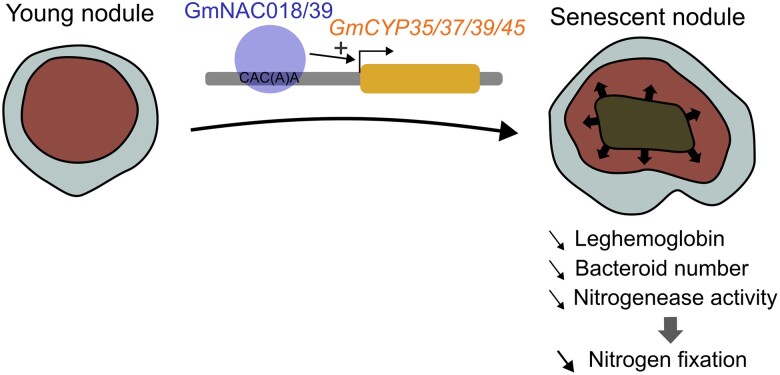
GmNAC018/GmNAC39 regulate nodule senescence by activating the *GmCYP35/GmCYP37/GmCYP39/GmCYP45* genes through direct binding on the CAC(A)A motif of their promoters. Nodule senescence results in decreased nitrogen fixation by the nodule. Arrows represent the progression of senescence that starts in the nodule center and spreads outwards. Created with Inkscape by N. M. Doll.

The authors then tried to unravel the gene regulatory network that triggers nodule senescence downstream of GmNAC039 and GmNAC018. By RNA-seq analyses they identified 5 genes encoding papain-like cysteine proteases (*CYPs*) that were upregulated both upon *GmNAC039* overexpression and during normal nodule senescence. *CYP*s are known to trigger cell death in other plant species and are thus interesting candidates for promoting nodule senescence (Buono et al 2019). Four of the 5 *CYP* genes identified, *GmCYP35/GmCYP37/GmCYP39/GmCYP45*, belong to the same subfamily and were investigated in more detail. All 4 *CYPs* were strongly downregulated in the *gmnac018 gmnac039* double mutant and were shown to be directly activated by GmNAC039, which binds the CAC(A)A core sequence present in their promoters. Overexpression of each of the 4 CYPs resulted in early nodule senescence, reminiscent of *GmNAC018* or *GmNAC039* overexpression. Conversely, the quadruple *gmcyp35 gmcyp37 gmcyp39 gmcyp45* knock-out mutant showed delayed nodule senescence, along with significantly higher nitrogenase activity in senescent nodules.

In summary, these results indicate that GmNAC018 and GmNAC039 promote nodule senescence through the direct activation of *GmCYP35/GmCYP37/GmCYP39/GmCYP45* genes (see Fig.). Loss of this pathway delays nodule senescence, which increases nitrogenase activity in older nodules. Future research should be conducted to analyze the impact of delayed nodule senescence on soybean growth and yield because it may cost energy for the plant to supply the bacteroids of the old nodules with carbohydrates. This result paves the way for better nitrogen fixation in legumes, a crucial step toward sustainable agriculture.
